# Clinical and digital assessment of tooth wear

**DOI:** 10.1038/s41598-023-50107-2

**Published:** 2024-01-05

**Authors:** Zahra Al-Seelawi, Nuno V. Hermann, Anne Peutzfeldt, Sara Baram, Merete Bakke, Liselotte Sonnesen, Angeliki Tsakanikou, Christos Rahiotis, Ana R. Benetti

**Affiliations:** 1 Section of Dental Materials, Department of Odontology, Faculty of Health and Medical Sciences, University of Copenhagen, Nørre Allé 20, DK-2200 Copenhagen, Denmark; 2 Section of Pediatric Dentistry and Clinical Genetics, Department of Odontology, Faculty of Health and Medical Sciences, University of Copenhagen, Copenhagen, Denmark; 3 Department of Preventive, Restorative and Pediatric Dentistry, School of Dental Medicine, University of Bern, Bern, Switzerland; 4 Section of Clinical Oral Physiology, Department of Odontology, Faculty of Health and Medical Sciences, University of Copenhagen, Copenhagen, Denmark; 5 Section of Orthodontics, Department of Odontology, Faculty of Health and Medical Sciences, University of Copenhagen, Copenhagen, Denmark; 6 Department of Operative Dentistry, School of Dentistry, National and Kapodistrian University of Athens, Athens, Greece

**Keywords:** Dental conditions, Dental equipment, Preventive dentistry

## Abstract

The aim of this study was to compare the assessment of tooth wear performed on digital models with the one conducted at the clinical examination. Seventy-eight volunteers (29 males and 49 females, age range 20–30 years) with at least 24 teeth, normal oral function, and a neutral transverse relationship were examined. During the clinical examination, dental wear was registered according to the Basic Erosive Wear Examination (BEWE) index. Subsequently, the BEWE index was blindly applied by two examiners on digital models obtained from the volunteers. Data were analyzed using weighted Cohen’s kappa coefficient and correlation tests with a confidence interval of 95%. All volunteers showed signs of tooth wear. Anterior teeth showed increased severity of tooth wear than first molars. Early loss of tooth substance could be identified on the digital models, including in areas with challenging direct intraoral visual access. Approximately 50% of the scores based on clinical examination agreed with those based on examination of digital models (*k* = 0.543, p < 0.01). A moderate, positive correlation was observed between scores registered clinically and on digital models (Spearman's rho = 0.560, p < 0.001). Considering the rather low agreement between the clinical and digital scores, alternatives to using BEWE on digital models are needed.

## Introduction

Tooth wear is an increasing challenge for global oral health. Recent epidemiological studies have shown a high prevalence of erosion in the permanent dentition of children, adolescents, and adults, up to as high as 88%^1,2^. A recent Swedish cross-sectional cohort study showed severe tooth wear to be as high as 28.3% among 15-year-olds and 34.3% among 17-year-olds^3^. Since the prevalence of dental erosion in adolescents is associated with lifestyle, diet, and sociodemographic and economic factors^1^, variation in the reported frequencies is expected due to differences in the investigated populations as well as in methodological differences within the studies.

The dentist’s ability to detect tooth wear at its earliest stage is crucial for implementing preventive measures and early onset of treatment, preventing later extensive and complex restorations, which are costly for the patient and challenging for the dentist^4^. The golden standard for detecting tooth wear is based on visual examination^5,6^. The teeth are inspected for any alteration in their morphology, and specific indices are employed to record the severity of the wear. Wear indices register tooth loss type and/or extent^5^: the type of tooth wear refers to the different wear mechanisms involved, whereas the extent refers to the degree of enamel loss and dentin exposure^6^. Therefore, a tooth wear index can be considered a tool for clinical examination, which, supplemented by clinical photographs and study models, may help registering and monitoring the severity of tooth wear^5^. Currently, as many as 114 different tooth wear index systems are defined in the literature^7^, the Basic Erosive Wear Examination (BEWE) being one of the most widely used indices today^6^.

Nevertheless, early recognition of initial tooth wear is not easy, and this condition is often not noted by the naked eye before a significant mineral loss has occurred. It is additionally challenging to visually detect premature tooth wear due to the lack of reference areas for comparison^8–10^, which contributes to the method’s low sensitivity in identifying tooth loss at its early stages^8^. This fact makes the quantification of tooth wear particularly important in estimating the wear progression rate, thereby preventing further tooth loss in the form of an early risk assessment and deciding on a preventive treatment plan^10^.

In vitro and in vivo studies have shown that a digital intraoral scanner system (IOS) is a promising technology for identifying and monitoring tooth wear^9,11^. However, the evaluation of the diagnostic advantages and limitations of using an IOS compared with the visual clinical examination is limited. This knowledge is essential for the successful implementation of an IOS-based assessment of tooth wear into clinical practice. Therefore, this study aimed to investigate the feasibility of using an intraoral scanner system for assessing the degree of tooth wear in young adults compared to the clinical examination in vivo.

## Material and methods

This cross-sectional study was carried out as a collaboration between the Department of Odontology, Faculty of Health and Medical Sciences, University of Copenhagen, Denmark and the Department of Operative Dentistry at the School of Dentistry, National & Kapodistrian University of Athens, Greece. The study was conducted in accordance with the declaration of Helsinki and the General Data Protection Regulation (GDPR). All included subjects participated voluntarily. This project is a quality control study, which according to the national regulation on Health Research Ethics, did not require approval from the national or regional Committee on Health Research Ethics. The clinical protocol was therefore approved by the local committee (A.U.TH. Research Committee protocol 474/14.10.2021). All participants signed an informed consent form including permission to use the data for research and publication in an anonymized form.

### Participants

Considering the assumption that 34.3% of the young adult population shows severe signs of tooth wear^3^, within the confidence level of 90% and margin of error of 10%, a minimum of 62 participants should be included in this study. Accounting for a possible 20% drop-out rate, a total of 78 subjects (29 males and 49 females) were included in the study (49 from the University of Copenhagen, Denmark, and 29 from the National & Kapodistrian University of Athens, Greece). The inclusion criteria were: (i) healthy adults without known general diseases, (ii) age range 20–30 years, (iii) at least 24 teeth, (iv) overjet and overbite between 1 and 5 mm, and (v) neutral transverse relationship of the dental arches on the posterior teeth. Exclusion criteria were severe malocclusion and current orthodontic treatment. Several patients (37%) reported having received previous orthodontic treatment with fixed appliances. Descriptive data are reported in Table 1.

**Table 1:** Descriptive information of the study population.

Information	Frequency, N (%)
**Age**	
20–30 years	78 (100%)
**Country of residence**	
Denmark	49 (63%)
Greece	29 (37%)
**Gender**	
Female	49 (63%)
Male	29 (37%)
**Earlier orthodontic treatment**	
None	48 (62%)
Fixed appliance	29 (37%)
Removable appliance	1 (1%)
**Right molar relation**	
Neutral occlusion	71 (91%)
1 Distal occlusion	3 (4%)
½ Distal occlusion	4 (5%)
Mesial occlusion	0 (0%)
**Left molar relation**	
Neutral occlusion	67 (86%)
1 Distal occlusion	3 (4%)
½ Distal occlusion	8 (10%)
Mesial occlusion	0 (0%)
**Transversal dental arch relation**	
Neutral	78 (100%)
Cross or reverse scissor bite	0 (0%)

### Clinical examination of tooth wear

After prophylaxis, the principal examiner (ZS) performed all the clinical visual assessment using standard illumination and a mirror to reflect the light onto the clean and dry tooth surfaces. The teeth were scored after calibration using the modified Basic Erosive Wear Examination (BEWE)^12^ criteria (Table 2). BEWE is a four-level scoring system where the appearance or severity of the wear on each tooth surface (facial, lingual, and occlusal/incisal) is assessed in relation to the total area of the respective surface. Only the highest score from the teeth in each tooth sextant was registered, starting from the upper right posterior teeth (sextant 1) and moving in a clockwise fashion until the lower right posterior teeth (sextant 6), while the cumulative score from all the sextants was recorded as the BEWE sum^12,13^. BEWE was not used on third molars, teeth with direct restorations covering more than 50% of the tooth surface area, or teeth with indirect restorations such as crowns or onlays.

**Table 2:** Description and illustration of modified BEWE scores.

Score	Intraoral photograph	Digital model (with colour texture)	Digital model (without colour texture)
BEWE 0 No sign of tooth wear.			
BEWE 1 Initial loss of the original enamel morphology affecting less than 25% of the visible occlusal tooth surface area.			
BEWE 2 Tooth loss affecting between 25 and 50% of the visible occlusal surface area. Flattening of the tooth surface is observed, dentine may be exposed.			
BEWE 3 Tooth loss affecting more than 50% of the occlusal tooth surface area. Visible, extended rounding of the teeth, presence of concavities, and dentine exposure.			

Representative images of the teeth on the intraoral photograph and the 3D digital models (with and without tooth color texture) from the same patient can be observed for each score. On the colour models, the area of tooth loss is outlined by a red stippled marking and cupping (i.e. concavity in dentine) by a black stippled marking.

### 3D intraoral scanning and examination of tooth wear on digital models

Immediately following the clinical examination, scanning of the participants’ upper and lower jaws, as well as digital registration of their occlusion, were performed with an intraoral scanner (IOS) (TRIOS 4, 3Shape Trios, Denmark) aided by software (TRIOS version 3.14.1.0 and Dental Desktop version. 1.7.25.1). Instructions from the manufacturer were followed during scanning; the operating lamp was switched off, the tooth surfaces had been professionally polished and air-dried, as mentioned earlier, and the recommended scan strategy was used^14^. After every scan, the “blue overlay” function was turned on to assess the scan quality based on the colour and fluorescence information. The teeth were scanned until the blue colour overlay cleared, which indicated sufficient information from the areas of interest (facial, lingual, and incisal/occlusal surfaces). The exact same intraoral scanner was employed in both centers, to guarantee that the same conditions were in place.

Calibration was performed before the proper assessment of all digital models. Two examiners (ZS, AB) blindly registered the BEWE scores twice on 30 randomly selected digital models at 2-week intervals without access to the clinical registrations or the other examiner's registrations. The inter- and intra-examiner agreement were calculated using weighted Cohens’s kappa coefficient. Inter- (p < 0.001, k ≥ 0.872) and intra-examiner reliability (p < 0.001, k_ZS_ = 0.844, k_AB_ = 0.795) were regarded as strong for the subsequent assessments.

Thereafter, the principal examiner (ZS) scored all digital models employing the modified BEWE criteria as previously described (Table 2). By combining views of the digital models with and without colour, different aspects of the tooth morphology were enhanced, which helped to determine the extent of the wear on the teeth.

### Statistical analysis

Statistical analysis was performed using the IBM SPSS Statistics tool (vers. 28.0.0.0). Descriptive analysis was extracted as frequencies for the categorical variables and median values for the scale variables. The agreement between the clinical BEWE scores and those applied to the digital models was calculated using weighted Cohen’s kappa coefficient. Cross-tabulation tables were created to illustrate the percentage of such agreement on a sextant level.

## Results

### Clinical BEWE scores

**Figure 1:**
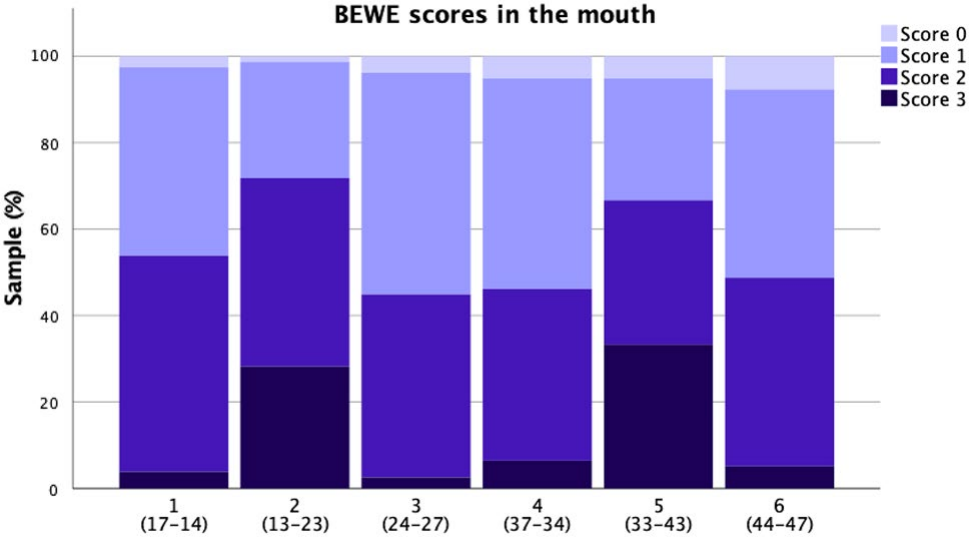
Distribution (%) of BEWE scores on a sextant level based on clinical examination. The corresponding examined teeth appear in parenthesis below each sextant number.

Figure 1 illustrates the distribution of BEWE scores in each sextant based on the clinical examination. Anterior teeth (sextants 2 and 5) showed the highest percentage of BEWE scores 3 (i.e. most severe wear) compared to the other sextants, in which scores 0 and 1 were dominant. As for the posterior teeth, there was a relatively even distribution between scores 2 and 1, and a markedly lower frequency of scores 3.

### Scores on digital models

**Figure 2:**
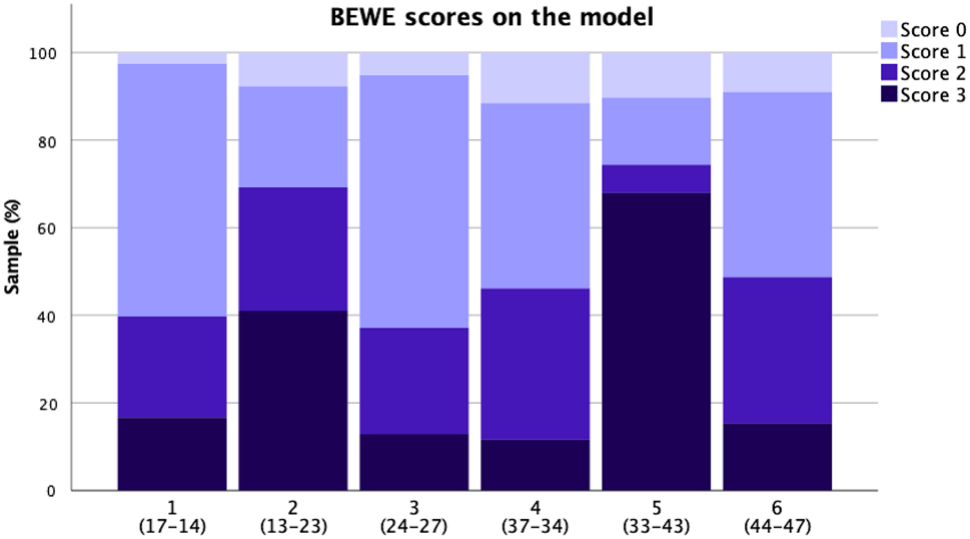
Distribution (%) of BEWE scores on a sextant level based on examination of digital models. The corresponding examined teeth appear in parenthesis below each sextant number.

Figure 2 shows the distribution of BEWE scores in each sextant of the digital models. The most severe wear (BEWE score 3) was seen on the anterior teeth (sextants 2 and 5). As for the posterior region, BEWE score 1 was given in approximately 50% of the cases, whereby the remaining percentages were distributed between scores of 2 and 3.

### Comparison of clinical BEWE scores and those given on the digital models

Considering all individual tooth scores, ratings on digital models agreed with those of the clinical examination in approximately 50% of cases (*k* = 0.543, p < 0.01); in 27% of cases, a higher score was given on the digital model and in 22% at the clinical examination. A moderate, positive correlation was observed between individual scores registered clinically and on digital models (Spearman's rho = 0.560, p < 0.001).

**Figure 3:**
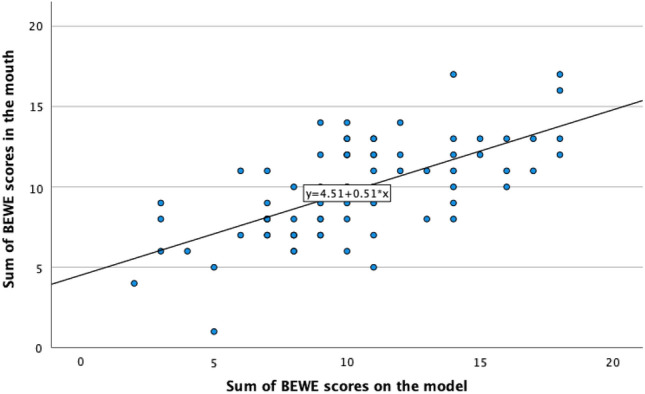
Correlation between the sum of BEWE scores based on clinical examination and on digital models (R^2^ linear = 0.421).

On a sextant level, the weighted kappa analysis showed a poor to moderate agreement between the BEWE scores attributed to the posterior teeth—clinically or on the models—for both the upper and lower jaws (Table 3). However, when the weighted kappa analysis was applied to the anterior teeth, the agreement was moderate for the upper jaw and fair for the lower jaw. Figure 3 shows the linear regression analysis between the BEWE sum registered clinically and on the digital models; a moderate, positive correlation (Pearson’s *r* = 0.649, p < 0.01) was found. See Supplementary Information for cross-tabulations between BEWE scores based on clinical examination and digital models.

**Table 3:** Cohen’s weighted kappa coefficient between clinical and digital scores per sextant and for BEWE sum.

	Sextant 1	Sextant 2	Sextant 3	Sextant 4	Sextant 5	Sextant 6	BEWE sum
Weighted kappa	0.163	0.482	0.270	0.325	0.417	0.345	0.382
Significance	< 0.041	< 0.001	< 0.001	< 0.001	< 0.001	< 0.001	< 0.001

## Discussion

An important finding in this study was that the 20–30-year-old patients showed more severe wear on the anterior teeth than on the first molars. Incisal edges of the anterior teeth showed a substantial loss of the enamel, often with the involvement of the dentin. Other studies have shown anterior teeth to be most affected by tooth wear^2,11^, which is partly because the incisors, after the first molars, are the teeth that have been in the mouth the longest^2^. Another explanation may be that it is easier to identify clinical wear on the anterior teeth. Additionally, teeth in the lower arch had a higher frequency of wear than the anterior teeth in the upper jaw. Earlier studies suggest that this can be explained by the fact that the enamel is thinner in the mandibular incisors than in the maxillary incisors^2,11^. This finding may also be caused by physiologic changes in the functional occlusion caused by the interplay between biomechanical factors and reciprocating adaptive responses, resulting in changes in masticatory patterns, and consequently tooth wear^15–17^. Other possible causes could be the consumption of acidic drinks, which mostly come into contact with the anterior teeth. Lastly, the wear pattern could have been influenced by previous malocclusion, which had been treated in 38% of the participants.

In a similar study from Finland, which also applied the BEWE index clinically and on digital models but on an older age group (mean 46 years), the anterior teeth similarly showed more severe wear than the first molars^11^. In contrast to the present study, the highest BEWE score registered in the anterior region in the Finnish study was 1. This discrepancy could be explained by the fact that the Finnish trial did not consider wear indicative of attrition alone (i.e. without an erosive component). Nonetheless, guidelines and recommendations of the BEWE index explain that this index can be used on all forms of tooth wear, and it is not limited to erosion^13^. Variation between the present and previous results reflect the degree of subjectivity of the BEWE index.

As the BEWE score per sextant results from the highest score given for any tooth in that region, this index is rather sensitive if one or few of the teeth deviate from the others. For example, extensively restored teeth, most often the first molars, are excluded from the rating, and the score for that sextant may thus be lower than expected. Other cases may be exempt from rating, such as mineralization defects, which may also have influenced the scoring. Another drawback of the BEWE index is that the extent (area) of wear is judged without necessarily considering the wear depth or level of dentin exposure. The BEWE index tends to underestimate the severity of wear if the affected area is small, but dentin exposure is present; for example, a single, minor cupping on a cusp (i.e. with dentin involvement) results in a BEWE score of 1, although in that location all enamel is lost. However, BEWE could be used to assess initial tooth wear and estimate the overall severity of tooth surface loss in the entire dentition.

The poorest agreement between the clinical BEWE scores and the digital model scores was found on the posterior teeth (molars). This finding was unexpected, as occlusal cuppings are one of the most prominent and easily detectable characteristics of acid damage-clinically and on digital models. The poor agreement may reflect difficulties in scoring the large and uneven occlusal surface area. Another problem may be the examiner’s unconscious tendency to assess the severity of wear in depth rather than considering only the affected area, potentially leading to a subjective score that goes beyond the BEWE guidelines. In most cases, a higher frequency of severe wear was registered on the digital models. These latter findings correspond with those from the Finnish study, probably reflecting that it is possible to detect even the most initial and small morphological changes on digital models compared to during the “true” clinical examination^11^. In the Finnish study, 6% of the participants were free of wear when the digital models were assessed, whereas 26% were wear-free in the clinical rating^11^. In the present study, 100% of the patients showed signs of tooth wear clinically or on digital models in at least one sextant.

Differences between the assessments performed clinically and on the digital models may be attributed to several factors. During the clinical examination, high-quality professional light enables the distinction between intact dental morphology and wear, as well as changes in the optical properties of the teeth. In particular, changes in the translucency of the enamel concerning the opacity of the underlying dentin due to enamel thinning are either not visible or very limited on the digital models. When applied to digital models, an important limitation of the BEWE index is that the transition between enamel and dentin cannot always be distinguished^12^. Additionally, the BEWE index is described very broadly, which opens the possibility for different interpretations from clinician to clinician. In fact, the inter-examiner reliability when BEWE index is applied has previously been reported to be as low as κ = 0.43^8^ or as high as κ = 0.87^11^. Such contrast in inter-examiner reliability scores exposes the fragility of the BEWE index. Another relevant consideration is the limited number of examiners participating in the current study. One may question whether allocating only two examiners for the assessment of digital models and one examiner for the clinical examinations is sufficient for a true representation of our findings. This is an important limitation of our study. Furthermore, our findings are based on digital models obtained using a specific intraoral scanner. As the technology, and thus quality of the acquired images, may vary by using different intraoral scanners, so may, in principle, the results.

The present study showed that capturing even the smallest wear facets on digital models was possible, as time is not a limiting factor compared to the clinical examination, and tooth surfaces are saliva-free. A key advantage of assessing wear on digital models is the possibility of adjusting their angulation, increasing their size, and observing them with or without colour texture. However, it is not always easy to distinguish a restoration from a natural tooth substance on digital models. In such cases, the clinical examination has the advantage of providing the natural optical properties of the tooth and the restoration, and the possibility to physically probe the site of interest. These observations correspond with those from the Finnish study^11^. It is the current authors’ experience that using the scanner was not difficult. Still, a systematic approach proved crucial for obtaining high-quality digital models. Also, in patients having difficulties opening their mouths, the use of an intraoral scanner may be problematic or even impossible.

## Conclusion

Assessment of tooth wear on digital models showed fair to moderate agreement with in vivo assessments. Our findings suggest that alternatives to using BEWE on digital models are worthy of further investigation.

### Supplementary Information

Supplementary Information.

## Data Availability

Data can be requested by contacting the corresponding author.
