# Supplementary material for: Clinical and digital assessment of tooth wear
Source: Sci Rep. 2024 Jan 5;14:592. doi: 10.1038/s41598-023-50107-2 (PMC10770026; doi:10.1038/s41598-023-50107-2)
Supplement: Supplementary file 1 — Supplementary Information. [file 41598_2023_50107_MOESM1_ESM.pdf]

Supplementary information

All cross-tabulations of the total number of the BEWE scores based on clinical examination vs. those based on the 3D models are provided as supplementary information. The green cells indicate agreement between the two scores. In the blue cells the scoring on the models was higher than the clinical score, while the opposite applies to the orange cells.

ANTERIOR TEETH

Upper Jaw

BEWE score in mouth		BEWE score on 3D model: 0	1	2	3	Total
	**0**	0	1	0	0	1
	**1**	5	11	4	1	21
	**2**	0	5	15	14	34
	**3**	1	1	3	17	22
Total		6	18	22	32	**78**

Agreement 43 55%   In vivo < model 20 26%   in vivo > model 15 19%

Lower Jaw

BEWE score in mouth		BEWE score on 3D model: 0	1	2	3	Total
	0	2	2	0	0	4
	1	6	7	1	8	22
	2	0	2	3	21	26
	3	0	1	1	24	26
Total		8	12	5	53	**78**

Agreement 36 46%   In vivo < model 32 41%   in vivo > model 10 13%

POSTERIOR TEETH (UPPER JAW)

In the left side

BEWE score in mouth	BEWE score on 3D model: 0	1	2	3	Total
0	0	1	1	1	3
1	4	29	5	2	40
2	0	15	12	6	33
3	0	0	1	1	2
Total	4	45	19	10	**78**

Agreement 42 54% In vivo < model 16 21% in vivo > model 20 26%

In the right side

BEWE score in mouth	BEWE score on 3D model: 0	1	2	3	Total
0	0	2	0	0	2
1	2	22	7	3	34
2	0	21	9	9	39
3	0	0	2	1	3
Total	2	45	18	13	**78**

Agreement 32 41% In vivo < model 21 27% in vivo > model 25 32%

POSTERIOR TEETH (LOWER JAW)

In the left side

BEWE score in mouth	BEWE score on 3D model: 0	1	2	3	Total
0	2	1	1	0	4
1	5	21	11	1	38
2	2	11	13	5	31
3	0	0	2	3	5
Total	9	33	27	9	**78**

Agreement 39 50% In vivo < model 19 24% in vivo > model 20 26%

In the right side

BEWE score in mouth	BEWE score on 3D model: 0	1	2	3	Total
0	2	1	3	0	6
1	4	21	7	2	34
2	1	10	16	7	34
3	0	1	0	3	4
Total	7	33	26	12	78

Agreement 42 54% In vivo < model 20 26% in vivo > model 16 21%
